# *Pseudomonas syringae* pv. *actinidiae* Effector HopAU1 Interacts with Calcium-Sensing Receptor to Activate Plant Immunity

**DOI:** 10.3390/ijms23010508

**Published:** 2022-01-03

**Authors:** Jinlong Zhang, Mingxia Zhou, Wei Liu, Jiajun Nie, Lili Huang

**Affiliations:** State Key Laboratory of Crop Stress Biology for Arid Areas, College of Plant Protection, Northwest A&F University, Xianyang 712100, China; zhangjinlong04@nwsuaf.edu.cn (J.Z.); zhoumingxia@nwsuaf.edu.cn (M.Z.); wliu@nwsuaf.edu.cn (W.L.); niejiajun@nwsuaf.edu.cn (J.N.)

**Keywords:** *Pseudomonas syringae* pv. *actinidiae*, effector, calcium-sensing receptor, resistance-related gene, plant immunity

## Abstract

Kiwifruit canker, caused by *Pseudomonas syringae* pv. *actinidiae* (*Psa*), is a destructive pathogen that globally threatens the kiwifruit industry. Understanding the molecular mechanism of plant-pathogen interaction can accelerate applying resistance breeding and controlling plant diseases. All known effectors secreted by pathogens play an important role in plant-pathogen interaction. However, the effectors in *Psa* and their function mechanism remain largely unclear. Here, we successfully identified a T3SS effector HopAU1 which had no virulence contribution to *Psa*, but could, however, induce cell death and activate a series of immune responses by agroinfiltration in *Nicotiana benthamiana*, including elevated transcripts of immune-related genes, accumulation of reactive oxygen species (ROS), and callose deposition. We found that HopAU1 interacted with a calcium sensing receptor in *N. benthamiana* (NbCaS) as well as its close homologue in kiwifruit (AcCaS). More importantly, silencing *CaS* by RNAi in *N. benthamiana* greatly attenuated HopAU1-triggered cell death, suggesting CaS is a crucial component for HopAU1 detection. Further researches showed that overexpression of *NbCaS* in *N. benthamiana* significantly enhanced plant resistance against *Sclerotinia sclerotiorum* and *Phytophthora capsici*, indicating that *CaS* serves as a promising resistance-related gene for disease resistance breeding. We concluded that HopAU1 is an immune elicitor that targets CaS to trigger plant immunity.

## 1. Introduction

Central to plant survival is the ability to activate immunity upon pathogen perception. Plant immunity is activated by sensing either conserved microbial signatures like pathogen-associated molecular patterns (PAMPs) or specific effectors secreted by pathogens [1]. To invade successfully, pathogenic bacteria secrete type III effectors into host cells to subvert plant immunity, and in turn, plants evolve towards effector recognition for enhancing immunity. Effector-triggered immunity (ETI) through intracellular receptors interferes with pathogen invasion early in host and converts it to rapid and robust defense [2]. During the course of their co-evolution, it increases the challenge of resistance breeding for preventing disease. Resistance breeding has been demonstrated to be one of the most efficient strategies for disease management. For disease-resistance breeding, the application of resistance-related genes is dramatically timesaving, compared with the conventional hybrids breeding approach [3]. Nonetheless, it remains a big challenge to obtain resistance-related genes before their successful application into breeding.

The exploitation of pathogen effectors to identify resistance genes is a powerful molecular tool for disease resistance breeding. Many plant resistance (R) genes have been successfully detected by effector-assisted strategies in numerous plants, such as tomato, rice and Arabidopsis [4]. As one of the most studied phytopathogens, *Pseudomonas syringae* has been demonstrated to employ T3SS effectors (T3SEs) as its core interaction with plants [5,6,7,8]. However, only several have been detected by plants, subsequently activating plant immune responses [9]. For example, AvrPtoB and AvrPto can physically interact with Pto kinase and elicit plant defenses in a Pto-dependent manner [10,11]. AvrB activates the JA signaling pathway by interacting with RIN4 [12,13]. In addition, three *Pseudomonas syringae* T3SEs (HopX1, HopZ1a and HopBB1) have been reported to target the COI1 receptor or JAZ transcriptional repressors to activate JA signaling [12,14,15,16]. As well as the aforementioned well-known effectors, additional effectors exhibit the ability to activate plant immunity, yet their direct resistance targets in plants are largely unknown (e.g., HopAS1, AvrRpt2 and AvrRps4) [17,18]. However, due to the diverse effector repertoires among *P. syringae* pathovars [19], the recognition mechanism between pathogens and plants remains to be elucidated.

The current pandemic *Pseudomonas syringae* pv. *actinidiae* biovar 3 (*Psa3*), the causal agent of the kiwifruit bacterial canker, has been recorded in all major kiwifruit cultivated areas since a sudden “2008 outbreak” in central Italy, and is a serious threat to global kiwifruit production [20,21]. *Psa*, as one of the most aggressive air-borne bacterial diseases, can severely infect aboveground kiwifruit organs (e.g., leaves, flower buds, twigs, and young shoots), restricting the healthy and sustainable development of the global kiwifruit industry [22,23,24,25]. Despite the forest plant quarantine measures applied worldwide to prevent an epidemic outbreak, persistent control strategies are still required [26]. Currently, great efforts have been made to reveal the *Psa* pathogenicity, yet the corresponding T3SEs and their functional mechanism are still unclear. Studies on *Psa* T3SEs have performed subcellular localization and transient expression to select several T3SEs triggered HR in *N. benthamiana* [27]. In addition, AvrE1*_Psa_* and HopR1*_Psa_* have been shown to be essential for full virulence, while HopM1 (belonging to the AvrE/HopM family) exhibits functional redundancy [28,29]. Additional research has focused on the recognition mechanism of *Psa* T3SEs in model plants. HopZ5*_Psa_*-tiggered ETI can be inhibited by SOBER1 in non-host Arabidopsis, while AvrRpm1*_Psa_* can be recognized by Rpa1 (but not RPM1) to trigger a plant immunity response [30,31,32,33]. Other than HopZ5 and AvrRpm1, reports of *Psa* T3SEs that can activate plant immune responses are limited. Moreover, the resistance-related genes in kiwifruit remain largely elusive, and the identification of these genes represents a big challenge.

Herein, we aim to employ HopAU1 for screening resistance-related genes. By bioinformatics and pathogenesis analysis, we demonstrate that HopAU1 is an evolutionarily conserved effector across *Pseudomonas* and make no contributions to *Psa* virulence. Using transient expression assays we demonstrate HopAU1 can induce HR-like cell death (HCD) and trigger immune response in *N.* *benthamiana*. Further evidence reveals that HopAU1 can interact with the host and nonhost CaS, which are responsible for HopAU1-induced cell death and, as a resistance-related gene, positively enhance plant immunity against phytopathogens in *N. benthamiana*. Taken together, our data reveals that HopAU1 act as an elicitor recognized by the resistance-related protein CaS to activate plant immunity.

## 2. Results

### 2.1. HopAU1 Is an Evolutionarily Conserved Effector in Pseudomonas

**Figure 1:**
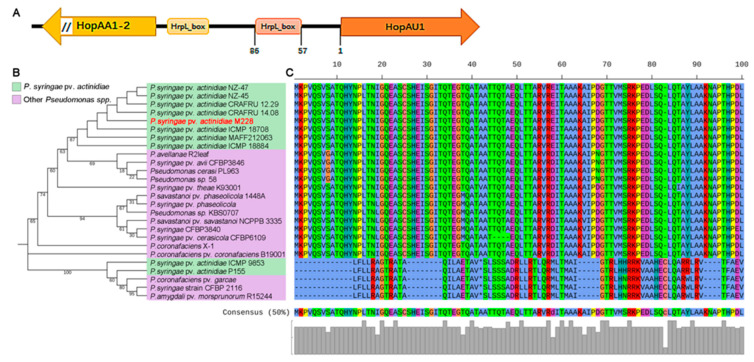
*HopAU1* homologues are conserved and widely distributed in *Pseudomonas*. (**A**) Gene structure characteristics of *HopAU1* display in plasmid genome of M228. (**B**) *HopAU1* and its homologues nucleic acid sequences were selected according to the BLAST results against to the NCBI-Nr database. The phylogeny tree was constructed using MEGA 7.0 software with the Neighbor-joining method. The data below bootstrap column support for each branch (gaps eliminated, 1000 bootstraps); *Pseudomonas syringae* pv. *actinidiae* and other *Pseudomonas* spp. are marked with light blue and purple background, respectively. (**C**) Multiple sequence alignment of HopAU1 homologues sequences directly next to the tree visualized by iTOL annotion (100 amino acid residues as sample).

*Psa* was observed to be a highly diverse group in terms of the genome arrangement of both phenotypes and genotypes, including many mobile genetic elements (e.g., IME and ICE, prophages, transposons and plasmids). For the high-virulence *Psa* strain M228 genome, *HopAU1* was identified as a single-copied gene encoding a protein of 731 amino acids and located in the plasmid with a consensus HrpL-mediated *hrp* box sequence in its upstream (Figure 1A). This indicates that *HopAU1* as a T3SE gene may transfer among *Pseudomonas syringaes* via plasmids.

For the construction of the *HopAU1* phylogenetic tree, 26 homologous sequences were selected against the National Center for Biotechnology Information (NCBI) database using the online BLASTN program. The results showed that *HopAU1* was widely distributed in various *Pseudomonas* spp., and mainly existed in *Psa* clusters, forming a close clade (Figure 1B). We then investigated the protein sequence conservation of 21 homologous HopAU1 sequences by selecting 100 amino acids as a representation of multiple sequence alignments. Apart from the pre-stop translation diverse clade, 21 homologous HopAU1 sequences exhibited more than 98% similarity consisting of the full-length protein alignment (Figure 1C and Figure S1). This suggests that *HopAU1* is widely distributed and evolutionarily conserved among *Pseudomonas* spp.

### 2.2. HopAU1 Makes No Contribution to Psa Virulence

**Figure 2:**
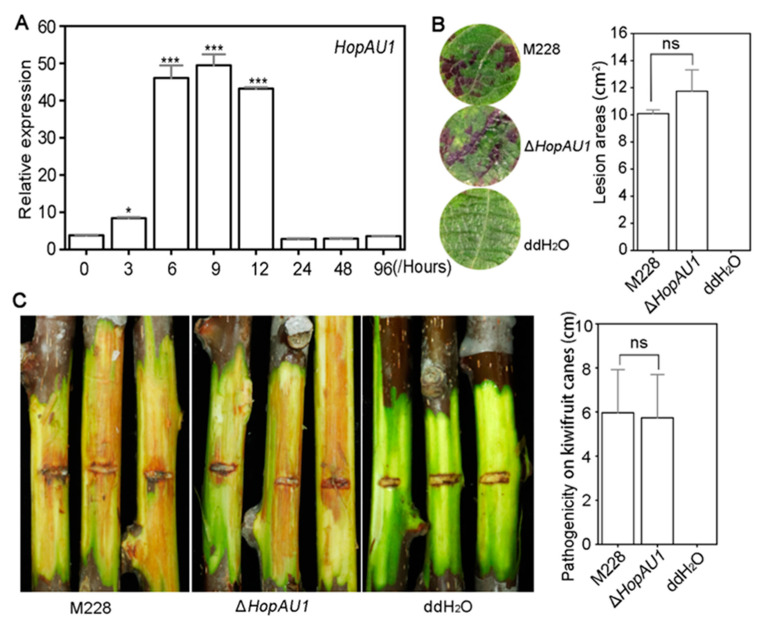
HopAU1 shows no obvious contribution to *Psa* M228 virulence on kiwifruit leaf dises and canes. (**A**), Relative expression level of HopAU1 in hrp-inducing media at 0, 3, 6, 12, 24, 48, and 96 h. The experiment repeats twice with similar results. (**B**), No significant differences in virulence were detected between M228 and Δ*HopAU1* post-inoculation on leaf discs of *Actinidiae chinensis* cv. HongYang. (**C**), No significant differences in virulence were detected between M228 and ΔhopAU1 post-inoculation on kiwifruit canes of *Actinidiae chinensis* cv. ‘HongYang’. The symptoms of B and C were observed at 5 days and 20 days post inoculation, respectively. For the inoculation, at least 15 canes or leaf discs were used for each treatment with three independent biological experiments. The statistical data were visualized with Mean ± standard errors (SEs) using Student’s *t*-test (***, *p* < 0.001; *, *p* < 0.05; ns, no significant, *p* > 0.05).

The relative expression level using qRT-PCR at eight different time point (0, 3, 6, 9, 12, 24, 48, and 96 h) showed that during the early inducing stage *HopAU1* was significantly up-regulated compared to its relative expression at 0 h. *HopAU1* was significantly up-regulated at 3, 6, 9 and 12 h, and its highest expression was observed at 9 h (fold change > 50) (Figure 2A). The expression pattern of *HopAU1* indicated is possible functionality during early infection.

In order to explore the virulence contribution of *HopAU1* in M228, we successfully obtained *HopAU1*-deletion mutant based on the homologous recombination method (Figure S2A). The virulence assays of the lesion areas did not reveal any significant differences between M228 and Δ*HopAU1* on kiwifruit leaf discs (Figure 2B). This was also true for the kiwifruit canes (Figure 2C). In order to facilitate further analysis of the *HopAU1* function in *Psa*, we constructed a 28 T3SEs-deletion mutant containing *HopAU1* in M228 denoted as M228Δ28E (unpublished). The single HopAU1-complement mutant M228Δ28E-C-*HopAU1* was then adopted to evaluate its virulence (Figure S2A,B). The virulence test assays did not reveal any significant differences between M228Δ28E-C-*HopAU1* and M228Δ28E-C-*GFP* on the kiwifruit leaf discs or canes (Figure S3A,B). These results demonstrate the limited influence of HopAU1 on the kiwifruit leaf discs and canes.

### 2.3. Transient Expression of HopAU1 Triggers Cell Death in N. benthamiana

**Figure 3:**
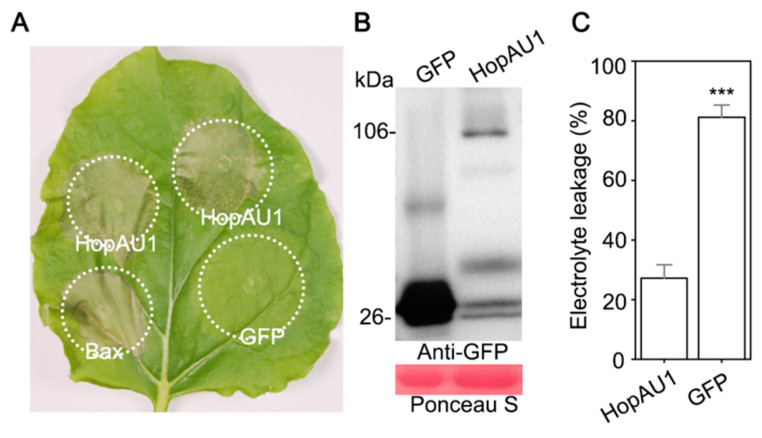
HopAU1 induces cell death in *Nicotiana benthamiana*. (**A**) HopAU1-induced cell death in *N. benthamiana*. The cell death phenomenon was photographed after 3 days in leaves of *N. benthamiana* with *Agrobacterium tumefaciens* carrying pCAMBIA1302-*HopAU1-GFP* as treatment, pCAMBIA1302-*Bax* and pCAMBIA1302-*GFP* as positive and negative control, respectively. (**B**) Western blotting analysis of proteins of *N. benthamiana* by agroinfiltration mediated transiently expression green fluorescent protein (GFP) control and HopAU1 tagged GFP. (**C**) Quantification of cell death by measuring electrolyte leakage after 3 days. The statistical data represent means ± standard errors (SEs) with three independent biological experiments; asterisks show significant differences between selected and EV for that assessment (***, *p* < 0.001; Student’s *t*-test).

The transient expression of *HopAU1* in *N. benthamiana* showed that HopAU1 triggered cell death after 3 days post-Agro-infiltration together with Bax. However, this was not true for the control GFP. The successful expression of *HopAU1* was further confirmed by western blotting. In addition, electrolyte leakage assays also demonstrated significant differences between HopAU1 and GFP (Figure 3A–C). Therefore, HopAU1 can induce HR-like cell death (HCD) in *N. benthamiana*.

### 2.4. HopAU1 Induces Immune Responses in N. benthamiana

**Figure 4:**
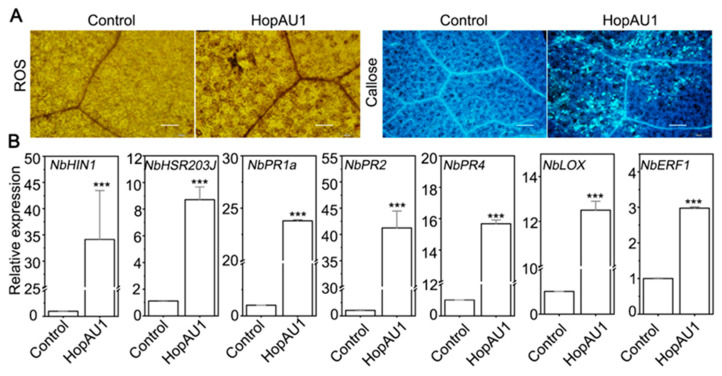
HopAU1 activates immunity responses in *Nicotiana* *benthamiana*. (**A**) ROS accumulation and callose deposition in *N. benthamiana* leaves by transiently expressing HopAU1-GFP and GFP control, respectively; Photographs were taken 2 days after infiltration; Bars = 50 μm. (**B**) qRT-PCR analysis of hypersensitive-response-speicfic marker genes and defense-related marker gene in *N. benthamina*. Transcript levels of candidate genes were normalized to that of *NbActin* and GFP was used as control. The statistical data were visualized with Mean ± standard errors (SEs) using Student’s *t*-test (***, *p* < 0.001).

The aforementioned *HopAU1* transiently expression in *N. benthamiana* leaves was sufficient to induce HR-like cell death 3 days post agroinfiltration. To test whether HopAU1-induced cell death had a relationship with plant immunity response, we examined the accumulation of reactive oxygen species (ROS) burst and callose deposition, and the transcript accumulation levels of two corresponding signal pathway marker genes, *HSR203J* and *HIN1* [34,35], in *N. benthamiana*. Figure 4A showed that ROS and callose were massive accumulated in *N. benthamiana* leaves after 2 days infiltration. Meanwhile, transcript accumulation levels of *HSR203J* and *HIN1* were significant up-regulated about 37-fold and 8-fold, respectively (Figure 4B). Those similar results indicate that HopAU1 was recognized by *N. benthamiana* and -triggered cell death related to plant HR immune response.

In order to further investigate whether *HopAU1* expression altered plant immune pathways, we detected the transcript accumulation levels of well-validated immune-related marker genes in *N. benthamiana*, including two vital marker genes of salicylic acid (SA)-dependent immunity, *NbPR1a* and *NbPR2* [36]; two marker genes of jasmonic acid (JA)-dependent immunity, *NbPR4* and *NbLOX* [37,38]; and one marker gene of ethylene-dependent, *NbERF1* [37,39]. As showed in Figure 4B, the relative expression levels of all selected immunity-related marker genes were up-regulated with varying degrees when transiently expression *HopAU1* after 2 days post agroinfiltration. These results demonstrate that HopAU1 acts as an elicitor that induces defense response in *N. benthamiana*.

### 2.5. HopAU1 Interacts with Plant Calcium Sensing Receptor (CaS)

**Figure 5:**
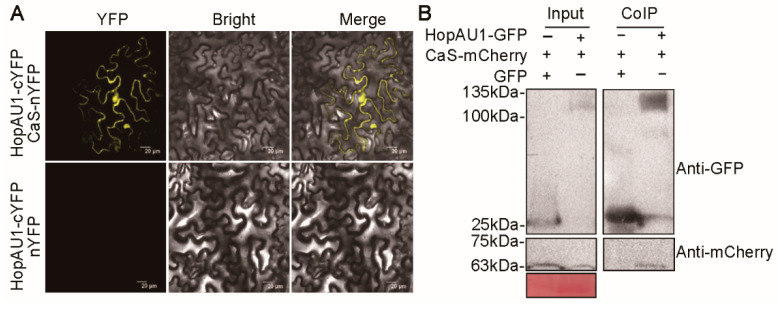
Confirmation of the interaction between HopAU1 and CaS. (**A**) BiFc analysis of HopAU1 and CaS in *N*. *benthamiana*. Photographs were taken after 2 days post HopAU1 tagged cYFP and CaS tagged nYFP co-expression in *N. benthamiana* leaves. Bars = 20 µm. (**B**) Co-immunoprecipitation (Co-IP) was performed for confirming the interaction. HopAU1-GFP and CaS-mCherry (or GFP and Cas-mCherry) were transiently co-expressed in *N. benthamiana*. Total protein was extracted and western blotting with anti-GFP or anti-mCherry antibodies, respectively. The bound proteins were affinity purified by GFP-Trap Immunomagnetic beads and then detected with the indicated antibodies.

In order to clarify the underlying mechanism between HopAU1 and the plant, we employed immunoprecipitation-mass spectrometry (IP-MS) to screen its candidate targets. In particular, HopAU1-GFP fusion protein was transiently expressed in *N. benthamiana* for 48 h and the bound proteins were then collected via GFP-Trap Immunomagnetic beads for IP-MS analysis. A total of 22 candidate targets were selected to identify the interaction relationship with HopAU1 (Supplementary Table S2). Large scale screening via by bimolecular fluorescent complementation (BiFC) and co-immunoprecipitation (CoIP) revealed that HopAU1 interacted with a calcium sensing receptor (CaS) in *N. benthamiana* (Figure 5A,B). In addition, the homologous protein AcCaS in kiwifruit could also positively interact with HopAU1 by CoIP (Figure S4). These results demonstrate the in-vivo and in-vitro interactions between HopAU1 and CaS.

### 2.6. CaS Is Required for HopAU1-Triggered Cell Death in N. benthamiana

**Figure 6:**
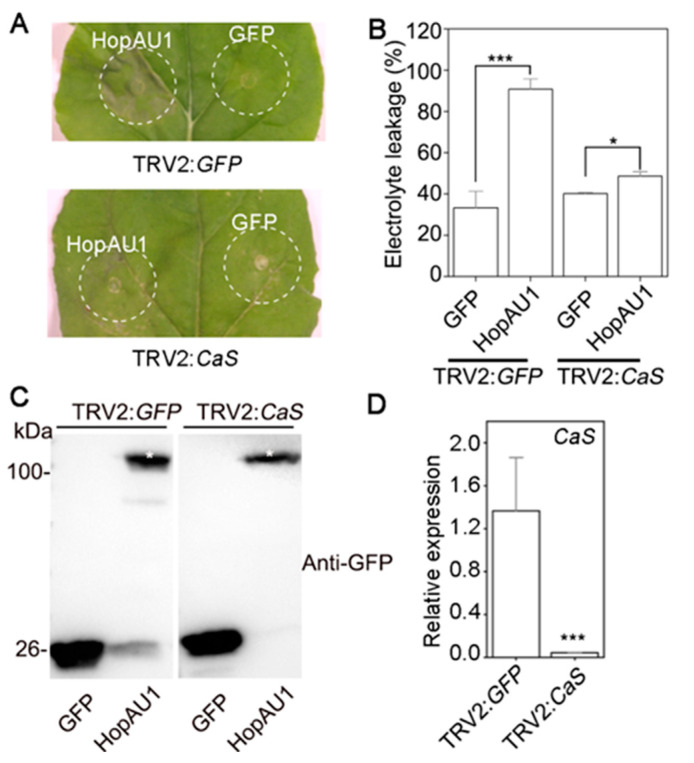
The *CaS*-silencing compromise HopAU1-induced cell death in *Nicotiana benthamiana*. (**A**) GV3101 containing *HopAU1-GFP* and *GFP* constructs were infiltrated into the region of the silenced *N. benthamiana* leaves. Cell death was recorded 3 days post infiltration. (**B**) Quantification of cell death was measured by electrolyte leakage. (**C**) *HopAU1-GFP* and *GFP* were transiently expressed in TRV-mediated silenced plant leaves. Immunoblot analysis the bounded proteins 2 days post inoculation. (**D**) The silencing efficiency was analyzed by qRT-PCR and photographs were taken at 3 weeks after viral inoculation. The *Nbactin* was used as the internal reference gene. Three biological repeats were performed in all experiments. Statistical data were visualized with Mean ± standard errors (SEs) using Student’s *t*-test (***, *p* < 0.001; *, *p* < 0.05).

To further determine whether CaS was required for HopAU1-triggered cell death, we generated VIGS (virus-induced gene silencing, VIGS) constructs to target the *CaS* expression in *N. benthamiana*. At 3 weeks after the VIGS treatments, the plants exhibited etiolation and dwarf phenotypes, and were then agroinfiltrated with GFP or HopAU1 (Figure S5). qRT-PCR analysis revealed the efficiency of *CaS*-silenced to fall to almost 95% compared with the control (Figure 6D). HopAU1 failed to trigger cell death in *CaS*-silenced *N. benthamiana*, yet it could trigger cell death in the control (Figure 6A,B). In addition, western blotting revealed that the transient expression of HopAU1 and GFP were successful in the corresponding silenced plants (Figure 6C). These results indicate CaS to be responsible for HopAU1-induced cell death in *N. benthamiana.*

### 2.7. Overexpression of CaS Mediates N. benthamiana Resistance to Sclerotinia Sclerotiorum and Phytophthora Capsici

**Figure 7:**
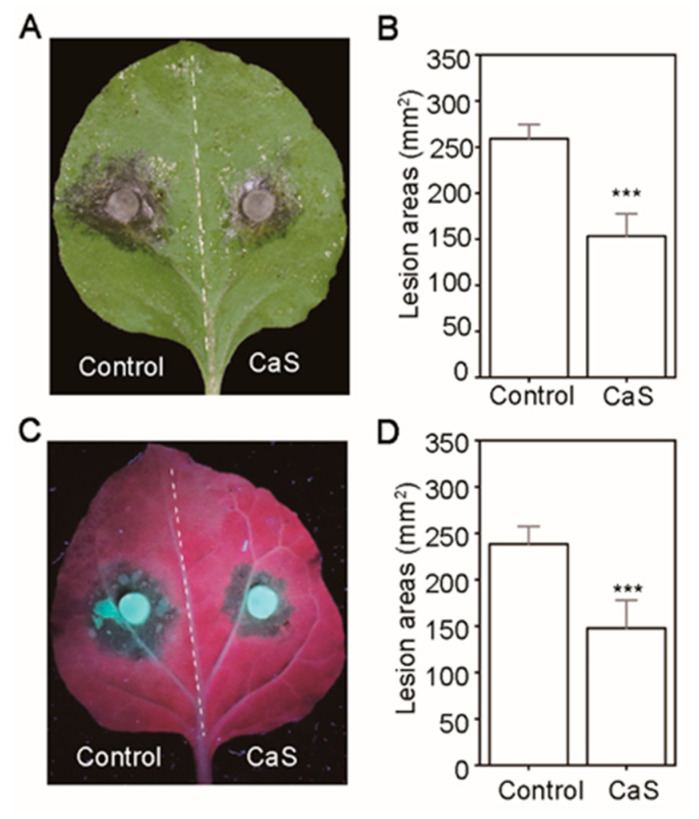
Overexpressing *CaS* enhances *N*. *benthamiana* resistance to *Sclerotinia sclerotiorum* and *Phytophthora capsici*. (**A**) *N. benthamiana* leaves were transiently expressed *GFP* (Left, control) and *HopAU1-GFP* (Right) 2 days before pathogen inoculation. Cell death was recorded at 24 h after inoculation of *S. sclerotiorum*. (**C**) Symptom of *N. benthamiana* leaves were photographed at 60 h after inoculation of *P. capsici*. (**B**,**D**) Lesion areas of *N. benthamiana* infected by *S. sclerotiorum* and *P. capsici* respectively, were calculated by ImageJ program. The experiment was conducted three biological repeats with at least ten technical replicates each and concluded with similar results. The statistical data was represented by Mean ± standard errors (SEs) using Student’s *t*-test (***, *p* < 0.001).

In order to further understand the CaS-influenced plant resistance, we generated the target protein *CaS* over-expression construct and inoculated a fungal pathogen and oomycete 3 days post-inoculation on the corresponding transient over-expression of *N. benthamiana* in leaves. The lesion areas were observed to be significantly attenuated following infection with the fungus *S. sclerotiorum* and the oomycete pathogen *P. capsici*, compared with the control (Figure 7A–D). This indicates the ability of CaS to enhance plant resistance against filamentous pathogen infection.

## 3. Discussion

The current pandemic *Pseudomonas syringae* pv. *actinidiae* biovar 3 (*Psa3*) has been identified as the most destructive disease to the global kiwifruit industry since 2008, causing bacterial organ diseases and cell deaths [20,21,40]. Thus far, only the monomorphic population *Psa3* has been reported in China, although it was known as divergence seriously, and biodiversity and genetic variation of population in destination countries [41,42,43]. This new aggressive phytopathogen directly delivers abundant T3SEs to interfere with host cellular processes and facilitate invasion. However, it is not clear how the T3SEs interacted with each other on the molecular level. Here, we identified a T3SE protein HopAU1, which could be recognized by the plants, and was conserved and widely distributed in *Pseudomonas* spp. However, the underlying interaction between HopAU1 and the plants was not clear.

HopAU1 is a plasmid-born protein. We predicted that HopAU1 was transferred from the mobile genetic element–namely, the plasmid–bringing new features to the *Psa*. In addition, a classical *hrp* box sequence was observed in the upstream of the *HopAU1* gene, targeted by the global regulator HrpL (Figure 1A), which was the key component of the HrpR/S-HrpL-T3SS/T3SEs hierarchical regulatory cascade in *P. syringae*. To further verify the *HopAU1* function in M228, we tested the virulence of Δ*HopAU1* and M228Δ28E-C-*HopAU1* via their inoculation on kiwifruit leaves and canes. The mutant pathogenicity did not exhibit any significant changes compared to the control, indicating that HopAU1 did not contribute to *Psa* virulence (Figure 2B,C and Figure S3A,B). This may be attributed to the possible role of HopAU1 as a no virulence protein in the M228 T3SE repertoire. More evidences showed that three T3SEs, HopR1, AvrE1 and HopZ5, contributed almost 80% to *Psa3* virulence [28,41], further supporting the no-virulence protein role of in *Psa*.

Furthermore, we used the *A. tumefaciens*-mediated transiently expression to examine the biological function of HopAU1 in *N. benthamiana*. In this study, HopAU1 was able to induce HR-like cell death and immunity responses, suggesting it could be recognized by the plants (Figure 3A,B). We then identified CaS from *N. benthamiana* or kiwifruit as the target protein interacting with HopAU1 in-vivo and in-vitro. Based on bioinformatics analysis, we determined the CaS gene to be located in the nucleus chromosome, which possessed a chloroplast transit peptide, indicating that CaS transcription occurred in nuclear, processed from Endoplasmic reticulum to Golgi apparatus, and eventually ended up in the chloroplast. Chloroplasts play an important role in the defense response plants and are a common target of the phytopathogen effectors [44]. HopI1 located in chloroplasts from *P. syringae* suppresses SA accumulation and leads to the reconstruction of the thylakoid structure [45]. HopN1, also from *P. syringae*, targets the chloroplasts PsbQ, suppressing the defense-associated ROS burst [46]. Moreover, the fungal and viral effectors target the chloroplasts to inhibit host resistance [47,48]. Our results suggest that HopAU1 triggers the immune response through interactions with CaS and subsequently affects the biological function of CaS. As we expected, the *HopAU1* transient expression in *CaS*-silenced *N. benthamiana* attenuated the cell death degree (Figure 6A,B). This indicates that CaS was specifically required for the HopAU1-triggered cell death. The CaS-mediated resistance response is associated with Ca^2+^ signaling which is able to cause programmed cell death (PCD) [47,48].Thus, the interaction between HopAU1 and CaS may assist CaS perception and transmission of calcium signaling eventually promoting plant cell death.

CaS is a Ca^2+^ sensing receptor involving modulation cytoplasmic Ca^2+^ concentrations and the regulation of the stomatal closure. However, the fine molecular function of CaS is unclear [49,50,51,52,53]. Previous studies have also demonstrated that CaS is required for defensive hormonal responses and anti-pathogens resistance [54,55]. For example, CaS positively regulates SA accumulation, ROS bursts, nuclear-encoded defense gene transcriptional reprogramming, and is required for PTI-induced and R gene-mediated ETI resistance [48]. Moreover, previous research has revealed that plant pathogens (e.g., the fungus *S. sclerotiorum* and the virus *Geminivirus* from different kingdoms) inhibit SA accumulation to facilitate infections by interacting with CaS [47,48,55]. Similar results in our study showed that the *CaS*-overexpression in *N. benthamiana* leaves restricted infection by filamentous pathogens compared with the control (Figure 7A–D), indicating CaS as a positive regulator of plant immunity to against the pathogens. These results further highlight the potential of CaS as a promising resistance-related protein in plant immunity responses.

## 4. Materials and Methods

### 4.1. Strains and the Growth of Plants

The *Psa* strain M228 was isolated from Mei County of Shaanxi province from infected leaves of *Actinidia chinensis* cv. Hongyang. The bacteria were grown in Luria-Bertani (LB) at 37 °C for *Escherichia coli* strain, 28 °C for *Agrobacterium tumefaciens* strain GV3101, and 25 °C for *Pseudomonas syringae* pv. *actinidiae* (*Psa*) containing appropriate antibiotics. All bacterial strains were stored in 25% glycerol solution at −80 °C. *N. benthamiana* seedlings were growing in a long-day glasshouse (14 h 22 °C:10 h 20 °C, day:night, 70% relative humidity).

### 4.2. Plasmid Construction

For Immunoblotting and confocal microscopy assays, *HopAU1* fragments were amplified from M228 genome, and *CaS* fragments from *N. benthamiana* cDNA and kiwifruit cDNA, respectively (gene-specific primers listed in Supplementary Table S1). Then the obtained gene fragments were separately ligated into pCAMBIA1302-GFP and PICH-mCherry (*Sp*eI and *Nc*oI for pCAMBIA1302-GFP, and *Cl*aI and *Sp*eI for PICH-mCherry), using ClonExpress II One-Step Cloning Kit (Vazyme, Nanjing, China). For constructing *HopAU1*-deletion recombinant vector, upstream and downstream flank of target genes separately amplified by PCR from M228 genome, and then cloned into the digested site of the suicide vector pK18*mobsacB* (The upstream and downstream flank sequence length is about 700 bp–1000 bp, *Eco*RI and *Hin*dIII for pK18mobsacB). For obtaining *HopAU1*-complement recombinant vector, *HopAU1* with a C-terminally tagged VSV-G was cloned into the digested site of the expression vector pDSK-GFPuv (*Nd*eI and *Bam*HI for pDSK-GFPuv) [56]. All constructs were confirmed by Sanger sequencing (Sangon Biotech, Shanghai, China). All mentioned primers in this study were listed in Supplementary Table S1.

### 4.3. Gene-Deletion and -Complement Mutants Generation

Gene-deletion mutants were based on the *SacB*-unmarked homologous replacement methods [57]. For obtaining *HopAU1*-deletion mutant of M228, the pK18*mobsacB* recombinant constructs were transferred from *E. coli* strain S17-1/λpir into M228 by conjugation transfer on LB agar for 2 days. Then, the transconjugants were selected on LB agar with appropriate antibiotics (nalidixic acid, 10 mg/L; ampicillin 10 mg/L; kanamycin, 50 mg/L) and confirmed by PCR (using primers SacB-F/R for the vectors and *Psa*-F/R for *Psa* stains). Next, the conjugants were performed to a counter-selection integration on 15% sucrose LB agar. Finally, the positive mutants were validated by PCR using detecting primer pairs (Supplementary Table S1). Following the *SacB*-based in-frame deletion method described above, M228Δ28E (unpublished, 28 T3SE genes were knocked out in M228 including HopAU1) and Δ*HopAU1* (single *HopAU1*-deletion in M228) mutants were finally obtained by largely screening (Figure S2A).

For obtaining complement mutants, the empty vector pDSK-GFPuv and carrying *HopAU1* were transformed into M228Δ28E by electroporation, respectively. Then, the complement mutants, M228Δ28E-C-*GFP* and M228Δ28E-C-*HopAU1*, were obtained for further pathogenicity analysis, and confirmed by Sanger sequencing (Figure S2B).

### 4.4. Bacterial Infiltrations for HR and Electrolyte Leakage Assays in N. benthamiana

The recombinant constructs were transformed into *A. tumefaciens* strain GV3101 by electroporation. For transient expression, *A. tumefaciens* strain GV3101 carrying HopAU1 was cultured in Luria-Bertani (LB) at 28 °C for 48 h, and then pelleted by centrifugation and resuspended in MES buffer (10 mM magnesium chloride (MgCl_2_), 10 mM MES, 200 µM acetosyringone, pH 5.7) in the dark for 3 h at room temperature (RT). Then, the 4-week-old *N. benthamiana* leaves was syringe infiltrated with *A. tumefaciens* strain adjusted to 4 × 10^8^ cfu/mL. Symptoms were evaluated by electrolyte leakage as described [58] and photographed after 3 days. *GFP* and *Bax* were used as negative and positive controls, respectively. All of the above experiments were repeated three times and produced similar results.

### 4.5. RNA Isolation, cDNA Synthesis, and Expression Analysis

Bacterial RNA extraction: The bacteria were grown in LB at 25 °C to OD_600_ of 0.2, harvested and washed three times adjust to OD_600_ of 0.5. Then the bacteria were transformed into *hrp*-derepressing minimal medium (HDM) for inducing expression by a ratio of 1:40. Samples were harvested, and total RNA of bacteria was extracted using RNApure Bacteria Kit (CWBIO, Jiangsu, China) according to the manufacturer’s protocol.

Plant RNA extraction: *N. benthamiana* leaves (4-week-old) were syringe infiltrated with bacterial strains. The infected leaves were sampled, frozen in liquid nitrogen and ground into power for RNA extraction. The total RNA was extracted by Quick RNA isolation Kit (Huayueyang, Beijing, China) following the manufacturer’s instructions.

All related cDNA were synthesized with 1ng of total RNA in a 20 μL reverse transcription mix (Thermo Scientific, Waltham, MA, USA) for next qRT-PCR assays on CFX connect Real-time System (Bio-Rad, Hercules, CA, USA). The *gyrB* [59,60] and the *NbActin* gene [61] was used as reference gene in *Psa* and *N. benthamiana* respectively. The relative expression level used was the 2^−ΔΔCT^ method, as described [62].

### 4.6. Confocal Microscopy and Co-Immunoprecipitation Assay (CoIP)

For BiFC assays, *HopAU1* and *CaS* were cloned into vector pBin61 forming recombination constructs (*HopAU1*-cYFP and nYFP-*CaS*) and then separately transformed into *A. tumefaciens* strain GV3101 by electroporation. The infected *N. benthamiana* leaves was photographed 3 days post-infiltration using a confocal laser scanning microscope (Olympus microscope FV1000, Tokyo, Japan) (YFP: excitation/emission wavelengths of 514 nm/530 nm-575 nm).

For CoIP assays, *HopAU1-GFP* and *CaS-mCherry* were transiently co-expressed in *N. benthamiana* and then were sampled for total protein extraction after 2 days. The same amounts of total proteins were incubated with pre-treated anti-GFP magnetic beads (ChromoTek GmbH, Munich, Germany) for 2 h at 4 °C according to the manufacturer’s instructions. Immunoprecipitates were analyzed by western blotting as described previously [63].

### 4.7. Callose and Reactive Oxygen Species (ROS) Staining

*N. benthamiana* leaves (4-week-old) were infiltrated with *A. tumefaciens* carrying recombinant constructs for transient expression. For callose staining, six leaf disks (1 cm diameter) were decolored by heating with 95% alcohol and then were placed in trypan blue liquid (0.01% trypan blue, 150 mM K_2_HPO_4_, pH 9.5) for 12 h overnight as described [64]. For H_2_O_2_ staining, the equated sampled were stained on diaminobezidin (DAB) liquid (1 mg/mL DAB, pH 3.8) at 12 h light and then decolored in 95% ethanol. All stained samples were observed and photographed using a confocal microscope (Olympus microscope FV1000, Tokyo, Japan). The experiments were repeated at least three times.

### 4.8. VIGS in N. benthamiana

For gene silencing assays, two lower leaves of two-week-old *N. benthamiana* were infiltrated with *A. tumefaciens* strain GV3101 containing pTRV1 and pTRV2 constructs. The infected plants were grown for 3 weeks before *A. tumefaciens* infection and HR assays. pTRV2:*PDS* and TRV2:*GFP* were used as controls and gene silencing level was confirmed by qRT-PCR.

### 4.9. Pathogenicity Tests

According to two indoor bioassay methods as previous described [41], vacuum infiltration and wound inoculation were used for pathogenicity tests. In experiments, at least fifteen kiwifruit leaf discs and canes were carried out for pathogenicity tests, and a replicated mock inoculation with sterile water was included as a negative control. The inoculated leaf discs and canes were incubated in a climate chamber at 16 h 18 °C:8 h 14 °C, day:night, 75% relative humidity. Analyses were repeated three times.

### 4.10. Accession Numbers and Bioinformatics Analysis

Sequence data accession nos. in this article was mentioned as follows. *HopAU1*_M228_ (CN228_RS32770, https://www.ncbi.nlm.nih.gov/, accessed on 18 February 2021), *NbCaS* (Niben101Scf18639g00026.1, https://solgenomics.net/, accessed on 6 April 2021), and *AcCaS* (Achn344161, http://kiwifruitgenome.org/, accessed on 6 April 2021).

For phylogenetic analysis, the *HopAU1* homologous sequences of different species were obtained from NCBI database by querying *HopAU1* nucleic acid sequence against Non-Redundant Protein/Nucleotide Sequence Database (Nr/nt), using the online BLASTN program (https://blast.ncbi.nlm.nih.gov/Blast.cgi, accessed on 18 February 2021), with default settings. Conserved domains of *HopAU1* were predicted by querying protein sequence against CDD v3.19-58,235 PSSMs database, using Batch CD-Search program (https://www.ncbi.nlm.nih.gov/cdd, accessed on 18 February 2021). The phylogenetic tree was constructed by the Mega7.0 with Neighbor-joining method, and beautified with tree annotation, colored ranges and multiple sequence alignments, by iTOL online website (https://itol.embl.de/itol_account.cgi, accessed on 15 August 2021).

## 5. Conclusions

Taken together, our results identify a conserved T3SE *HopAU1* distributing across various *Pseudomonas* spp., which can trigger plant immunity response. More studies reveal the interaction between HopAU1 and CaS in *N. benthamiana* or kiwifruit. Here, we successfully employ the effector HopAU1 to screen resistance-related protein CaS, as a resistance resource, which is expected to be used for molecular resistance breeding against plant pathogens in the future.

## Data Availability

All data supporting the findings of this study are provided in the manuscript and its Supplementary Files. Additional data supporting the findings of this study are available from the corresponding authors upon request.

## Supplementary Materials

The following supporting information can be downloaded at: https://www.mdpi.com/article/10.3390/ijms23010508/s1.
