# Supplementary material for: Pseudomonas syringae pv. actinidiae Effector HopAU1 Interacts with Calcium-Sensing Receptor to Activate Plant Immunity
Source: Int J Mol Sci. 2022 Jan 3;23(1):508. doi: 10.3390/ijms23010508 (PMC8745740; doi:10.3390/ijms23010508)
Supplement: Supplementary file 1 [file ijms-23-00508-s001.zip › ijms-1501293-supplementary.pdf]

International Journal of
Molecular Sciences

MDPI

Supplementary Materials

Pseudomonas syringae pv. *actinidiae* Effector HopAU1 Interacts with Calcium-Sensing Receptor to Activate Plant Immunity

Jinlong Zhang, Mingxia Zhou, Wei Liu, Jiajun Nie and Lili Huang *

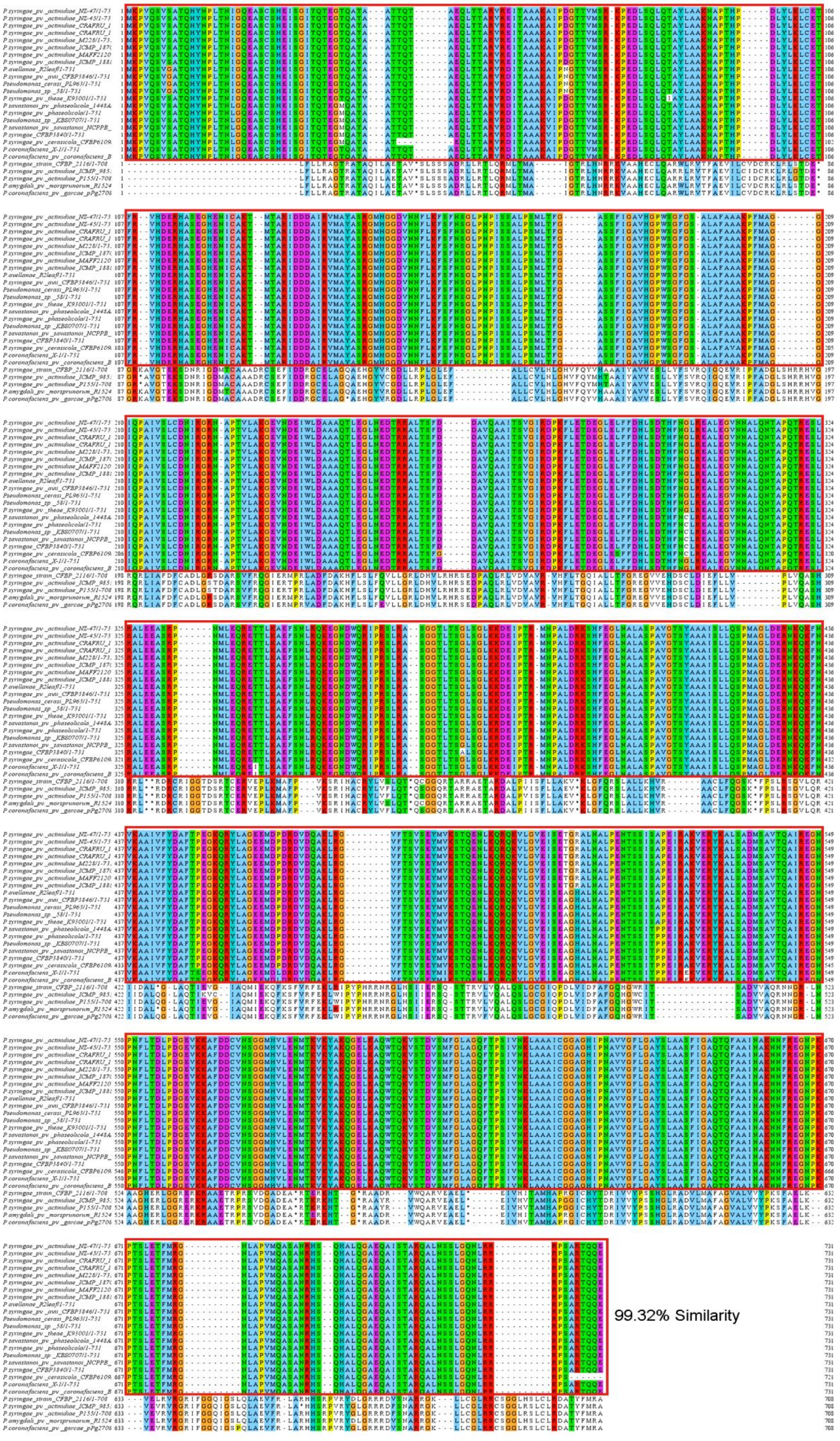
99.32% Similarity

Figure S1. Multiple sequence alignment of HopAU1 homologues sequence based on full-length protein. 26 homologues sequence was aligned by ClustalW program, and beautified by software Jalview 2.11.1.5 version.

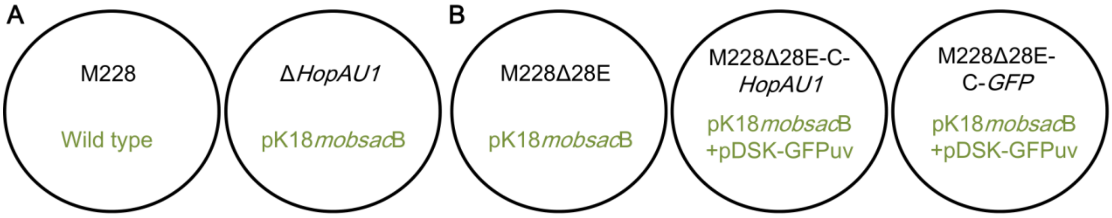

Figure S2. Schematic diagram of the mutants and their required plasmids. (A), *Psa* M228 was the high-virulent wild-type strain. The pK18*mobsac*B recombinant construct was used for generate the Δ*HopAU1* mutant based on the wild type M228; (B), The pK18*mobsac*B recombinant constructs were used to generate M228Δ28E, in which 28 T3SEs genes (including *HopAU1*) were all deleted. The pDSK-GFPuv or its recombinant constructs carrying *HopAU1* were used to generate the complement mutants, M228Δ28E-C-*GFP* and M228Δ28E-C-*HopAU1*, respectively. Black words represent the different strain; Blue words represent the recombinant constructs for different mutants.

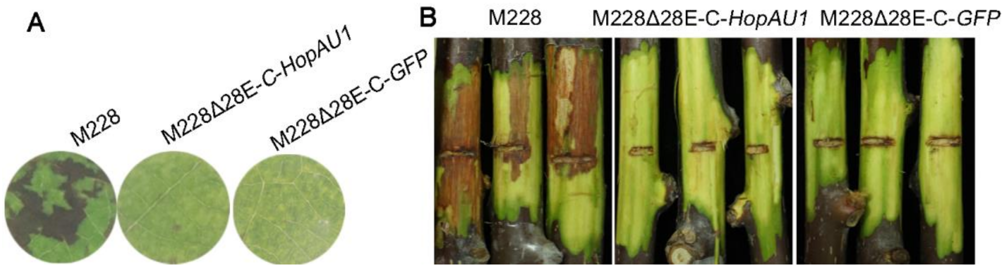

Figure S3. *HopAU1*-complement had no virulence contribution to *Psa* M228. **(A)**, Vacuum infiltration inoculation on leaf discs with 10^4 cfu/ml, at 5 dpi. HopAU1 induced no cell death on the leaf discs of *Actinidiae chinensis* cv. 'HongYang'. **(B)**, Wound inoculation on detached 'HongYang' canes with 10^8 cfu/ml bacteria, observed at 15 days post inoculation (dpi). M228 was the high-virulent *Pseudomonas syringe* pv. *actinidiae* (Psa) strain, as the positive control; M228Δ28E-*C-HopAU1* as *HopAU1*-complement strain; M228Δ28E-C-*GFP*, a *GFP*-complement mutant, as the negative control. For the inoculation, at least 10 canes or leaf discs were used for each strain.

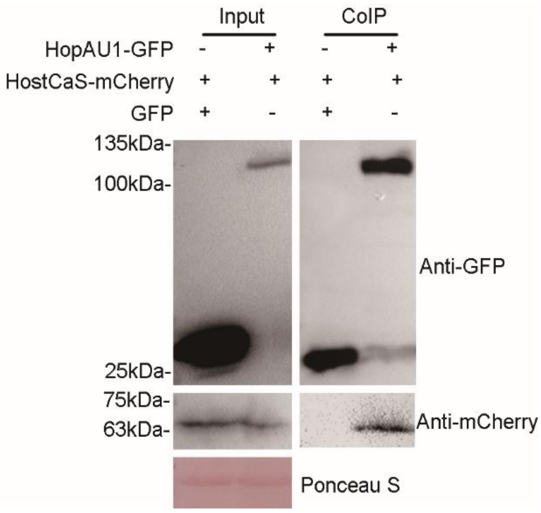

Figure S4. Co-immunoprecipitation analysis the interaction of HopAU1 and the kiwifruit protein CaS. Total proteins were extracted from *N. benthamiana* leaves co-expressing with HopAU1-GFP and HostCaS-mCherry (or GFP and HostCaS-mCherry as control), respectively. The total extracted and the affinity purified proteins were validated by using the indicated antibodies immunblot.

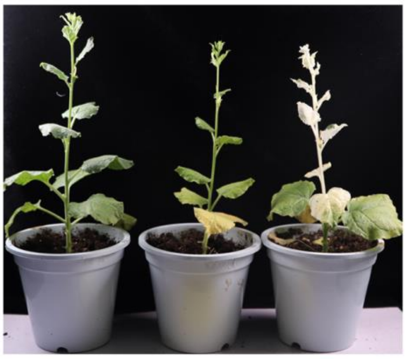

Figure S5. Silencing of CaS inhibits the growth of *N. benthamiana.* Two-week-old *N. benthamiana* plants were used for gene silencing by agroinfiltration of tobacco rattle virus (TRV) constructs (TRV2:*GFP*, TRV2:*CaS*, TRV2:*PDS*). Upper leaves of the silencing plants were used for testing silencing efficiency 3 weeks post infiltration. pTRV2:*PDS* and TRV2:*GFP* were used as positive and negative controls, respectively. The experiment was conducted three times with six TRV-mediated silenced plants.

Table S1. Primers used in this study.

Primer Name	Sequence	Purpose	Purpose
pDSK-HopAU1-F	GAAGGAGATATACATATGAAGCCCGTTCAATCAGTTAGT	Construct HopAU1-complement vector	
pDSK-HopAU1-R	GACTCTAGAGGATCCTTACTTACCCAGGCGGTTCATTTCGATATCAGTG-TATTCCTGCTGCGTGCGGGC		
pK18-HopAU1-EcoRI-UP-F	CTATGACATGATTACGAATTCGCAGCGGCAAATGGAGTGTT		
pK18-HopAU1-UP-R	CAATCAGCGCCGACATCTCA		
pK18-HopAU1-DOWN-F	CGGCGCTGATTGTAGGGTCTGTTCCCGTTTCAC		
pK18-HopAU1-HindIII-DOWN-R	ACGACGGCCAGTGCCAAGCTTGCTCGCAGCACATGACAAAT		
PSA-F	CAGAGGCGCTAACGAGGAAA	Genaration HopAU1-deletion mutant in M228	
PSA-R	CGAGCATACATCAACAGGTCA		
SacB-F	GCAAACACTGGAACTGAAGATGG		
SacB-R	TTCCTTTCGCTTGAGGTACAGC		
qRT-HopAU1-F	CGTGTTCTACGACGCCTTCA		
qRT-HopAU1-R	CCCGTCTCGCTGATTTCG		qRT-PCR analysis HopAU1 in M228
qRT-gyrB-F	ACCCGAACGAAGCCAAAGC		
qRT-gyrB-R	ATCCGCCAGCAGAGTCCC		
cYFC-HopAU1-XbaI-F	GCCCAAGCTTCGACTCTAGAATGAAGCCCGTTCAATCAGTTAGT		
cYFC-HopAU1-BamHI-R	ACGCTGCCGTCCATGGATCCTTCCTGCTGCGTGCGGGC	BiFc analysis in N. benthamiana	
nYFP-NbCaS-BamHI-F	GCCACAACATCGAGGGATCCATGGCGCTTAGAGCTTCAGC		
nYFP-NbCaS-SmaI-R	TTCGAGCTCTATCCCGGGTTAATCACTACCCCCTGAAAGCA		
pCAMBIA1302-HopAU1-GFP-NcoI-F	GGGGACTCTTGACCATGGTAATGAAGCCCGTTCAATCAGTTAGT		
pCAMBIA1302-HopAU1-GFP-SpeI-R	CTCACCATCCTAGGACTAGTTTCCTGCTGCGTGCGGGC		
PICH-HopAU1-mCherry-ClaI-F	CATTTACAATTATCGATATGAAGCCCGTTCAATCAGTTAGT		
PICH-HopAU1-mCherry-SpeI-R	CTCACCCTAGGACTAGTTTCCTGCTGCGTGCGGGC		
pCAMBIA1302-NbCaS-GFP-NcoI-F	GGGGACTCTTGACCATGGTAATGGCGCTTAGAGCTTCAGC	Transiently exressing in N. benthamiana	
pCAMBIA1302-NbCaS-GFP-SpeI-R	CTCACCATCCTAGGACTAGTATCACTACCCCCTGAAAGC		
PICH-NbCaS-mCherry-ClaI-F	CATTTACAATTATCGATATGGCGCTTAGAGCTTCAGC		
PICH-NbCaS-mCherry-SpeI-R	CTCACCCTAGGACTAGTATCACTACCCCCTGAAAGC		
pCAMBIA1302-AcCaS-mCherry-NcoI-F	CATTTACAATTATCGATATGGCCGTAGGAGTCTCTGCC		

pCAMBIA1302-AcCaS-mCherry-SpeI-R	CTCACCCTAGGACTAGTATCAGAGCGCCCCGGGA	
TRV-Cas-BamHI-F	GTGAGCTCGGTACCGGATCCATGGACGCTCAACCAGTGATG	
TRV-Cas-EcoRI-R	GAGTAAGGTTACCGAATTCCTCTGTTCTAATATCAATCAAGACATAATT	
qRT-NbActin-F	TGGTCGTACCACCGGTATTGTGTT	qRT-PCR analysis for VIGS efficiency
qRT-NbActin-R	TCACTTGCCCATCAGGAAGCTCAT	
qRT-NbCaS-F	GAGAAGGACAAGGATAAGGCTGG	
qRT-NbCaS-R	CCTTTGTTGATTTTCTTGAGGTATG	
NbActin-F	TGGTCGTACCACCGGTATTGTGTT	
NbActin-R	TCACTTGCCCATCAGGAAGCTCAT	
qRT-NbHIN1-F	CCAACTTGAACGGAGCCTATTA	
qRT-NbHIN1-R	AGGCATCCAAAGAGACAACTAC	
qRT-NbHSR203J-F	ACGCAGATTTCAACCGAGTAT	
qRT-NbHSR203J-R	GCCAGTCGCATTGGAGATAA	
qRT-NbPR1a-F	CCGCCTTCCCTCAACTCAAC	
qRT-NbPR1a-R	GCACAACCAAGACGTACTGAG	qRT-PCR analysis in N. benthamiana
qRT-NbPR2-F	AGGTGTTTGCTATGGAATGC	
qRT-NbPR2-R	TCTGTACCCACCATCTTGC	
qRT-NbPR4-F	GGCCAAGATTCCTGTGGTAGAT	
qRT-NbPR4-R	CACTGTTGTTTGAGTTCCTGTTCCT	
qRT-NbLOX-F	AAAACCTATGCCTCAAGAAC	
qRT-NbLOX-R	ACTGCTGCATAGGCTTTGG	
qRT-NbERF1-F	GCTCTTAACGTCGGATGGTC	
qRT-NbERF1-R	AGCCAAACCCTAGCTCCATT	

Table S2. 22 candidate targets of HopAU1 selected from mass spectrometry (IP-MS).

Accession	Description
Niben101Scf01326g09009.1 sp	Q5M786 WDR5_XENTR *-*- WD repeat-containing protein 5 IPR001810 (F-box domain), IPR015943 (WD40/YVTN repeat-like-containing domain) GO:0005515 (protein binding)
Niben101Scf03804g05014.1	AT4G08310.1 ***- FUNCTIONS IN: molecular_function unknown; INVOLVED IN: biological_process unknown; LOCATED IN: cellular_component unknown; EXPRESSED IN: 25 plant structures; EXPRESSED DURING: 13 growth stages; IPR009057 (Homeodomain-like), IPR019098 (Histone chaperone domain CHZ) GO:0003677 (DNA binding)
Niben101Scf18639g00026.1	AT5G23060.1 ***- calcium sensing receptor LENGTH=387 IPR001763 (Rhodanese-like domain)
Niben101Scf00860g03018.1	AT5G51100.1 **** Fe superoxide dismutase 2 LENGTH=305 IPR001189 (Manganese/iron superoxide dismutase) GO:0004784 (superoxide dismutase activity), GO:0006801 (superoxide metabolic process), GO:0046872 (metal ion binding), GO:0055114 (oxidation-reduction process)
Niben101Scf00693g02022.1 sp	Q1CZI7 HTPG_MYXXD *-*- Chaperone protein HtpG IPR001404 (Heat shock protein Hsp90 family) GO:0005524 (ATP binding), GO:0006457 (protein folding), GO:0006950 (response to stress), GO:0051082 (unfolded protein binding)
Niben101Scf07242g07006.1	AT3G28740.1 ***- Cytochrome P450 superfamily protein LENGTH=509 IPR001128 (Cytochrome P450) GO:0005506 (iron ion binding), GO:0016705 (oxidoreductase activity, acting on paired donors, with incorporation or reduction of molecular oxygen), GO:0020037 (heme binding), GO:0055114 (oxidation-reduction process)
Niben101Scf01453g06010.1 sp	Q96520 PER12_ARATH **** Peroxidase 12 IPR010255 (Haem peroxidase) GO:0004601 (peroxidase activity), GO:0006979 (response to oxidative stress), GO:0020037 (heme binding), GO:0055114 (oxidation-reduction process)
Niben101Scf00117g02019.1 sp	B2S0M0 DNAK_BORHD ***- Chaperone protein DnaK IPR013126 (Heat shock protein 70 family), IPR029047 (Heat shock protein 70kD, peptide-binding domain), IPR029048 (Heat shock protein 70kD, C-terminal domain) GO:0005524 (ATP binding), GO:0006457 (protein folding), GO:0051082 (unfolded protein binding)
Niben101Scf05389g00010.1 sp	P34930 HS71A_PIG *-*- Heat shock 70 kDa protein 1A IPR013126 (Heat shock protein 70 family), IPR029047 (Heat shock protein 70kD, peptide-binding domain), IPR029048 (Heat shock protein 70kD, C-terminal domain)
Niben101Scf05688g08010.1	AT1G56190.1 **** Phosphoglycerate kinase family protein LENGTH=478 IPR001576 (Phosphoglycerate kinase) GO:0004618 (phosphoglycerate kinase activity), GO:0006096 (glycolytic process)

Niben101Scf23814g00038.1 sp	P59259 H4_ARATH *-*- Histone H4 IPR001951 (Histone H4), IPR009072 (Histone-fold) GO:0000786 (nucleosome), GO:0003677 (DNA binding), GO:0005634 (nucleus), GO:0006334 (nucleosome assembly), GO:0006352 (DNA-templated transcription, initiation), GO:0046982 (protein heterodimerization activity)
Niben101Scf00031g02001.1 sp	Q9FLV4 Y5248_ARATH *-*- G-type lectin S-receptor-like serine/threonine-protein kinase IPR001480 (Bulb-type lectin domain)
Niben101Scf02461g00004.1	AT1G56190.1 **** Phosphoglycerate kinase family protein LENGTH=478 IPR001576 (Phosphoglycerate kinase) GO:0004618 (phosphoglycerate kinase activity), GO:0006096 (glycolytic process)
Niben101Scf00069g12017.1	AT5G26780.3 **** serine hydroxymethyltransferase 2 LENGTH=533 IPR001085 (Serine hydroxymethyltransferase), IPR015424 (Pyridoxal phosphate-dependent transferase) GO:0003824 (catalytic activity), GO:0004372 (glycine hydroxymethyltransferase activity), GO:0006544 (glycine metabolic process), GO:0006563 (L-serine metabolic process), GO:0030170 (pyridoxal phosphate binding)
Niben101Scf04361g01001.1 sp	P37218 H1_SOLLC *-*- Histone H1 IPR005819 (Histone H5) GO:0000786 (nucleosome), GO:0003677 (DNA binding), GO:0005634 (nucleus), GO:0006334 (nucleosome assembly)
Niben101Scf02461g00004.1	AT1G56190.1 **** Phosphoglycerate kinase family protein LENGTH=478 IPR001576 (Phosphoglycerate kinase) GO:0004618 (phosphoglycerate kinase activity), GO:0006096 (glycolytic process)
Niben101Scf00117g02019.1 sp	B2S0M0 DNAK_BORHD ***- Chaperone protein DnaK IPR013126 (Heat shock protein 70 family), IPR029047 (Heat shock protein 70kD, peptide-binding domain), IPR029048 (Heat shock protein 70kD, C-terminal domain) GO:0005524 (ATP binding), GO:0006457 (protein folding), GO:0051082 (unfolded protein binding)
Niben101Scf05389g00010.1 sp	P34930 HS71A_PIG *-*- Heat shock 70 kDa protein 1A IPR013126 (Heat shock protein 70 family), IPR029047 (Heat shock protein 70kD, peptide-binding domain), IPR029048 (Heat shock protein 70kD, C-terminal domain)
Niben101Scf03631g00005.1 sp	O24339 CATA_SOLAP **** Catalase IPR002226 (Catalase haem-binding site), IPR010582 (Catalase immune-responsive domain), IPR011614 (Catalase core domain), IPR018028 (Catalase, mono-functional, haem-containing), IPR020835 (Catalase-like domain), IPR024708 (Catalase active site) GO:0004096 (catalase activity), GO:0006979 (response to oxidative stress), GO:0020037 (heme binding), GO:0055114 (oxidation-reduction process)
Niben101Scf06685g00010.1 sp	Q43695 TBB3_MAIZE ***- Tubulin beta-3 chain IPR000217 (Tubulin), IPR023123 (Tubulin, C-terminal) GO:0003924 (GTPase activity), GO:0005200 (structural constituent of cytoskeleton), GO:0005525 (GTP binding), GO:0005874 (microtubule), GO:0006184 (GTP catabolic process), GO:0007017 (microtubule-based process), GO:0043234 (protein complex), GO:0051258 (protein polymerization)
Niben101Scf05824g12005.1 sp	O22348 TBA2_ELEIN ***- Tubulin alpha-2 chain IPR000217 (Tubulin), IPR023123 (Tubulin, C-terminal) GO:0003924 (GTPase activity), GO:0005200 (structural constituent of cytoskeleton), GO:0005525 (GTP binding), GO:0005874 (microtubule), GO:0006184 (GTP catabolic process), GO:0007017 (microtubule-based process), GO:0043234 (protein complex), GO:0051258 (protein polymerization)

Niben101Scf04328g00049.1 sp	B1A930 PSBD_CARPA *-** Photosystem II D2 protein IPR000484 (Photosynthetic reaction centre, L/M), IPR000932 (Photosystem antenna protein-like) GO:0009521 (photosystem), GO:0009767 (photosynthetic electron transport chain), GO:0009772 (photosynthetic electron transport in photosystem II), GO:0016020 (membrane), GO:0016168 (chlorophyll binding), GO:0019684 (photosynthesis, light reaction), GO:0045156 (electron transporter, transferring electrons within the cyclic electron transport pathway of photosynthesis activity)
Niben101Scf01326g09009.1 sp	Q5M786 WDR5_XENTR *-*- WD repeat-containing protein 5 IPR001810 (F-box domain), IPR015943 (WD40/YVTN repeat-like-containing domain) GO:0005515 (protein binding)
Niben101Scf03804g05014.1	AT4G08310.1 ***- FUNCTIONS IN: molecular_function unknown; INVOLVED IN: biological_process unknown; LOCATED IN: cellular_component unknown; EXPRESSED IN: 25 plant structures; EXPRESSED DURING: 13 growth stages; IPR009057 (Homeodomain-like), IPR019098 (Histone chaperone domain CHZ) GO:0003677 (DNA binding)
Niben101Scf18639g00026.1	AT5G23060.1 ***- calcium sensing receptor LENGTH=387 IPR001763 (Rhodanese-like domain)
Niben101Scf00860g03018.1	AT5G51100.1 **** Fe superoxide dismutase 2 LENGTH=305 IPR001189 (Manganese/iron superoxide dismutase) GO:0004784 (superoxide dismutase activity), GO:0006801 (superoxide metabolic process), GO:0046872 (metal ion binding), GO:0055114 (oxidation-reduction process)
Niben101Scf00693g02022.1 sp	Q1CZI7 HTPG_MYXXD *-*- Chaperone protein HtpG IPR001404 (Heat shock protein Hsp90 family) GO:0005524 (ATP binding), GO:0006457 (protein folding), GO:0006950 (response to stress), GO:0051082 (unfolded protein binding)
Niben101Scf07242g07006.1	AT3G28740.1 ***- Cytochrome P450 superfamily protein LENGTH=509 IPR001128 (Cytochrome P450) GO:0005506 (iron ion binding), GO:0016705 (oxidoreductase activity, acting on paired donors, with incorporation or reduction of molecular oxygen), GO:0020037 (heme binding), GO:0055114 (oxidation-reduction process)
Niben101Scf01453g06010.1 sp	Q96520 PER12_ARATH **** Peroxidase 12 IPR010255 (Haem peroxidase) GO:0004601 (peroxidase activity), GO:0006979 (response to oxidative stress), GO:0020037 (heme binding), GO:0055114 (oxidation-reduction process)
Niben101Scf00117g02019.1 sp	B2S0M0 DNAK_BORHD ***- Chaperone protein DnaK IPR013126 (Heat shock protein 70 family), IPR029047 (Heat shock protein 70kD, peptide-binding domain), IPR029048 (Heat shock protein 70kD, C-terminal domain) GO:0005524 (ATP binding), GO:0006457 (protein folding), GO:0051082 (unfolded protein binding)
Niben101Scf05389g00010.1 sp	P34930 HS71A_PIG *-*- Heat shock 70 kDa protein 1A IPR013126 (Heat shock protein 70 family), IPR029047 (Heat shock protein 70kD, peptide-binding domain), IPR029048 (Heat shock protein 70kD, C-terminal domain)
Niben101Scf05688g08010.1	AT1G56190.1 **** Phosphoglycerate kinase family protein LENGTH=478 IPR001576 (Phosphoglycerate kinase) GO:0004618 (phosphoglycerate kinase activity), GO:0006096 (glycolytic process)
Niben101Scf23814g00038.1 sp	P59259 H4_ARATH *-*- Histone H4 IPR001951 (Histone H4), IPR009072 (Histone-fold) GO:0000786 (nucleosome), GO:0003677 (DNA binding), GO:0005634 (nucleus), GO:0006334 (nucleosome assembly), GO:0006352 (DNA-templated transcription, initiation), GO:0046982 (protein heterodimerization activity)

Niben101Scf00031g02001.1 sp	Q9FLV4 Y5248_ARATH *-*- G-type lectin S-receptor-like serine/threonine-protein kinase IPR001480 (Bulb-type lectin domain)
Niben101Scf02461g00004.1	AT1G56190.1 **** Phosphoglycerate kinase family protein LENGTH=478 IPR001576 (Phosphoglycerate kinase) GO:0004618 (phosphoglycerate kinase activity), GO:0006096 (glycolytic process)
Niben101Scf00069g12017.1	AT5G26780.3 **** serine hydroxymethyltransferase 2 LENGTH=533 IPR001085 (Serine hydroxymethyltransferase), IPR015424 (Pyridoxal phosphate-dependent transferase) GO:0003824 (catalytic activity), GO:0004372 (glycine hydroxymethyltransferase activity), GO:0006544 (glycine metabolic process), GO:0006563 (L-serine metabolic process), GO:0030170 (pyridoxal phosphate binding)
Niben101Scf04361g01001.1 sp	P37218 H1_SOLLC *-*- Histone H1 IPR005819 (Histone H5) GO:0000786 (nucleosome), GO:0003677 (DNA binding), GO:0005634 (nucleus), GO:0006334 (nucleosome assembly)
Niben101Scf02461g00004.1	AT1G56190.1 **** Phosphoglycerate kinase family protein LENGTH=478 IPR001576 (Phosphoglycerate kinase) GO:0004618 (phosphoglycerate kinase activity), GO:0006096 (glycolytic process)
Niben101Scf00117g02019.1 sp	B2S0M0 DNAK_BORHD ***- Chaperone protein DnaK IPR013126 (Heat shock protein 70 family), IPR029047 (Heat shock protein 70kD, peptide-binding domain), IPR029048 (Heat shock protein 70kD, C-terminal domain) GO:0005524 (ATP binding), GO:0006457 (protein folding), GO:0051082 (unfolded protein binding)
Niben101Scf05389g00010.1 sp	P34930 HS71A_PIG *-*- Heat shock 70 kDa protein 1A IPR013126 (Heat shock protein 70 family), IPR029047 (Heat shock protein 70kD, peptide-binding domain), IPR029048 (Heat shock protein 70kD, C-terminal domain)
Niben101Scf03631g00005.1 sp	O24339 CATA_SOLAP **** Catalase IPR002226 (Catalase haem-binding site), IPR010582 (Catalase immune-responsive domain), IPR011614 (Catalase core domain), IPR018028 (Catalase, mono-functional, haem-containing), IPR020835 (Catalase-like domain), IPR024708 (Catalase active site) GO:0004096 (catalase activity), GO:0006979 (response to oxidative stress), GO:0020037 (heme binding), GO:0055114 (oxidation-reduction process)
Niben101Scf06685g00010.1 sp	Q43695 TBB3_MAIZE ***- Tubulin beta-3 chain IPR000217 (Tubulin), IPR023123 (Tubulin, C-terminal) GO:0003924 (GTPase activity), GO:0005200 (structural constituent of cytoskeleton), GO:0005525 (GTP binding), GO:0005874 (microtubule), GO:0006184 (GTP catabolic process), GO:0007017 (microtubule-based process), GO:0043234 (protein complex), GO:0051258 (protein polymerization)
Niben101Scf05824g12005.1 sp	O22348 TBA2_ELEIN ***- Tubulin alpha-2 chain IPR000217 (Tubulin), IPR023123 (Tubulin, C-terminal) GO:0003924 (GTPase activity), GO:0005200 (structural constituent of cytoskeleton), GO:0005525 (GTP binding), GO:0005874 (microtubule), GO:0006184 (GTP catabolic process), GO:0007017 (microtubule-based process), GO:0043234 (protein complex), GO:0051258 (protein polymerization)
Niben101Scf04328g00049.1 sp	B1A930 PSBD_CARPA *-** Photosystem II D2 protein IPR000484 (Photosynthetic reaction centre, L/M), IPR000932 (Photosystem antenna protein-like) GO:0009521 (photosystem), GO:0009767 (photosynthetic electron transport chain), GO:0009772 (photosynthetic electron transport in photosystem II), GO:0016020 (membrane), GO:0016168 (chlorophyll binding), GO:0019684 (photosynthesis, light reaction), GO:0045156 (electron transporter, transferring electrons within the cyclic electron transport pathway of photosynthesis activity)

MS sequence obtained by BLAST against Nicotiana benthamiana genome (https://solgenomics.net/)
